# Introduction and overview of offenders with intellectual disabilities

**DOI:** 10.1192/j.eurpsy.2024.104

**Published:** 2024-08-27

**Authors:** K. Goethals

**Affiliations:** ^1^University Forensic Centre, Antwerp University Hospital, Edegem; ^2^CAPRI, University of Antwerp, Wilrijk, Belgium

## Abstract

**Abstract:**

Intellectual disability (according to the DSM-5) or intellectual developmental disorder (according to the ICD-11) is a disorder with onset during the developmental period that includes both intellectual and adaptive functioning deficits in conceptual, social and practical domains. The term learning disability (LD) is also used, although this term shows more specifically deficits in the domain of learning. The term learning difficulties is often used for specific or generalized intellectual impairment that does not meet all of the criteria of LD.

The prevalence of learning disability in prisoners is about 10%. Up to 60% of male prisoners have learning difficulties. Prevalence rates for offending behaviour in patients with LD is higher than in the general population and show a large range, from 2-40%.

The main explanatory factor underlying the link between intelligence and offending is the lack of ability to manipulate abstract concepts. Poor academic performance, common in persons with LD, is also linked to offending.

With regard to sexual offending, some persons with LD may not have learnt the rules that define acceptable and unacceptable behaviour. Sexual offences may amount to inappropriate, impulsive expressions of emotion rather than premediated violent acts. Violent behaviour in the LD population may be due to frustration, impulsivity or poor problem solving skills. There is no significant difference in the frequency of violent or property offences between individuals with LD and those without. However, sex offences and fire-setting are frequently seen in individuals with LD.

Persons with LD are vulnerable suspects and may also be disadvantaged by the criminal justice system because of a lack of appropriate support and legal representation from early stages in the process.

In this introductory paper these themes will be addressed.

**Disclosure of Interest:**

None Declared

